# Clinical Algorithm for the Management of Ipsilateral Proximal and Shaft Femur Fractures Using Single or Dual Implants

**DOI:** 10.7759/cureus.55359

**Published:** 2024-03-01

**Authors:** Deepak Rai, Ajit Singh, Gautam Tripathi

**Affiliations:** 1 Orthopedic Surgery, Institute Of Medical Sciences (IMS) Banaras Hindu University, Varanasi, IND; 2 Orthopedic Surgery, Banaras Hindu University, Varanasi, IND

**Keywords:** single implant, dual implant, concomitant, long proximal femoral nail, ipsilateral fracture hip and shaft femur

## Abstract

Purpose of the study

The current study had two goals: first, it compared the radiological and functional results of the ipsilateral shaft and proximal femur fractures treated by using two different methods, i.e., single implant vs dual implants. The second goal was to devise a clinical algorithm for guiding and managing such fractures.

Methods

This study was conducted in a level 1 trauma center and included 34 patients with concomitant ipsilateral fractures of the proximal femur and shaft of the femur. The patients were divided into two groups as per our clinical algorithm. Group I, comprising of 16 patients, were treated with a single implant like the proximal femoral nail (PFN) or proximal femoral nail antirotation (PFNA2). Group II of dual implants, comprising of 18 patients, were treated with two types of implants separately for proximal and shaft fracture.

Results

All patients were followed at monthly intervals up to six months, then at three monthly intervals up to one year, with a minimal follow-up of one year of every patient. On clinical evaluation by Friedman-Wyman criteria, in group I, seven patients had a fair outcome, eight patients had a good outcome, and one patient had a poor outcome, while in group II, eight patients had a fair outcome, nine patients had a good outcome, and one patient had a poor outcome. No patient developed non-union or avascular necrosis of the femoral head in any of the groups.

Conclusion

For concurrent ipsilateral diaphyseal and proximal femur fractures, both dual and single implants are equally effective alternatives if properly applied as per our clinical algorithm. Implant selection primarily depends on the pattern of injury, and our clinical algorithm can be a suitable guide for guiding the selection of implants.

## Introduction

A rare pattern of injuries, ipsilateral proximal femur and shaft femur fractures occur for 2.5-9% of femur fractures [1-3]. The majority are encountered in high-energy trauma, such as road traffic accidents and falls from heights [1,3-6]. Treatment of ipsilateral proximal femur and shaft femur fractures is technically challenging and complicated, and there are several surgical methods available. To treat these difficult fractures, many procedures and implants have been devised [3,5,7,8]. However, there is no agreement on how best to treat them [1-3]. Each method has its own merits and demerits. All the previously published literature on this challenging fracture pattern is based on a retrospective review of a few cases [9-13].

Basically, these treatment modalities can be divided into two groups: a) single implant - in which one implant takes care of both fractures, and b) dual implants - in which two implants are used, which allow the individualized treatment of each fracture. The purpose of this study was to compare the functional outcome of concomitant ipsilateral fractures of the proximal femur and shaft of the femur treated by using two different methods, i.e., single implant vs dual implants. This is the first prospective study to devise a clinical algorithm for guiding the management of such difficult fractures.

## Materials and methods

**Figure 1:**
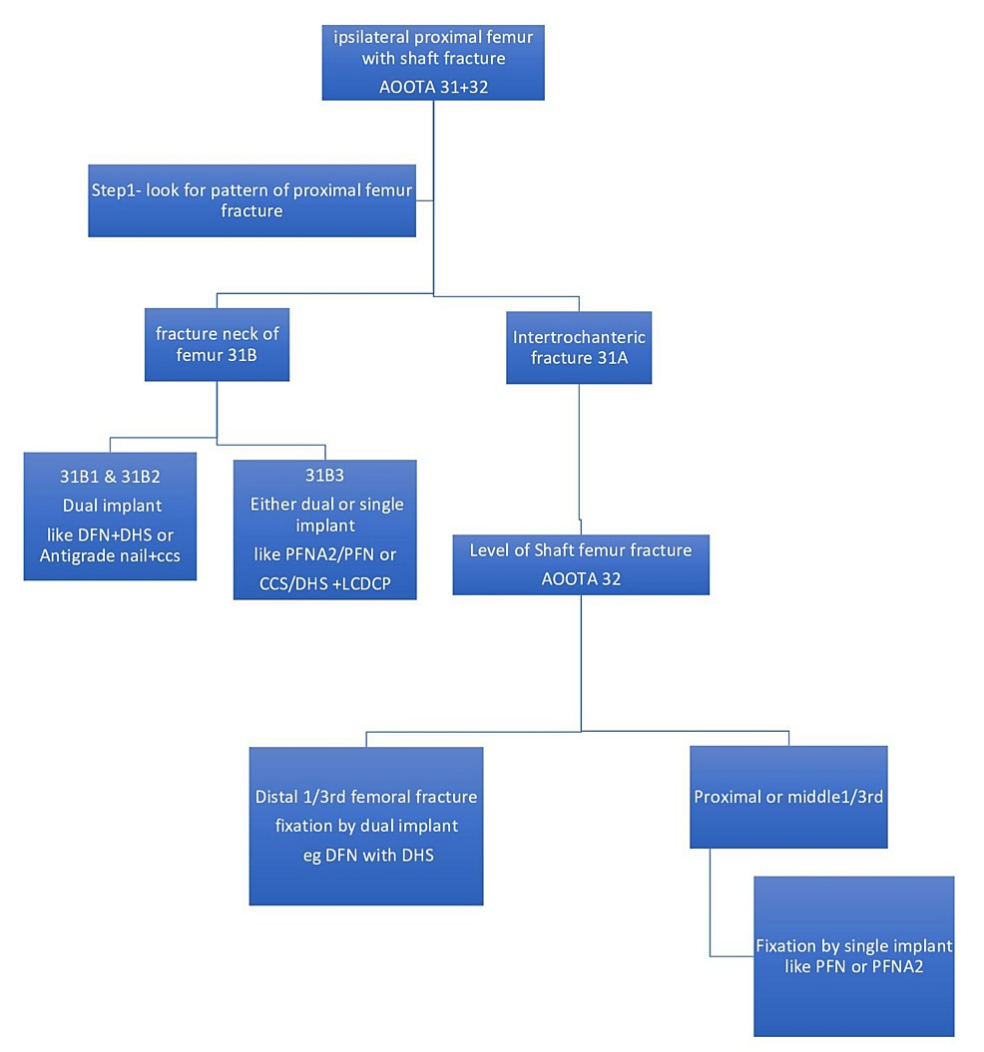
Clinical algorithm for deciding management of a combination of ipsilateral proximal and shaft of femur fractures DFN - distal femoral nail; DHS - dynamic hip screw; CCS - cannulated cancellous screws; PFN - proximal femoral nail; PFNA2 - proximal femoral nail antirotation; LCDCP - limited contact dynamic compression plate

This institutional-based prospective comparative study was conducted in the Department of Orthopedics of the level 1 trauma center of a tertiary care university teaching hospital, Banaras Hindu University, after approval of the Institutional Ethical Committee. Between April 2016 to May 2019, 34 patients with concomitant ipsilateral fractures of the proximal femur and shaft of the femur were registered for the study after giving informed written consent for the study protocol. After a discussion with the operating team at our institute and a review of previous studies [14-21], a management plan/algorithm was made (Figure 1). The patients were divided into two groups.

Group I, comprising of 16 patients, were treated with a single implant like a proximal femoral nail (PFN) or proximal femoral nail antirotation (PFNA2).

Group II, comprising of 18 patients, was treated with dual implants like a combination of plate/distal femoral nail (DFN) with dynamic hip screw (DHS)/cannulated cancellous screws (CCS).

Patients were advised to do passive range of motion (ROM) exercises and walk with partial weight-bearing for six weeks following surgery, and progressive weight-bearing was allowed until radiological union. All patients were followed at monthly intervals up to six months, then three monthly intervals up to one year, with a one-year minimum follow-up for each patient. The follow-up study included both a functional Friedman-Wyman scoring system [2] (Table 1) and radiological evaluations. Any change in fracture union progress was recorded and documented.

**Table 1:** Friedman-Wyman scoring system

Grade	Perturbation of daily activity	Pain	Lost of motion of hip or knee
Good	No perturbations	None	<20%
Average	Average	Average to moderate	20-50%
Fair	Moderate	Severe	>50%

Statistical analysis

Analysis of the data was conducted with the help of SPSS version 25 (IBM Inc., Armonk, New York). The descriptive analysis (e.g. mean and standard deviation) was done for normally distributed parameters and their means were compared using the Chi-squared test with Yates' correction and Student's t-test. For all tests, p-value <0.05 was considered to be significant.

## Results

**Figure 2:**
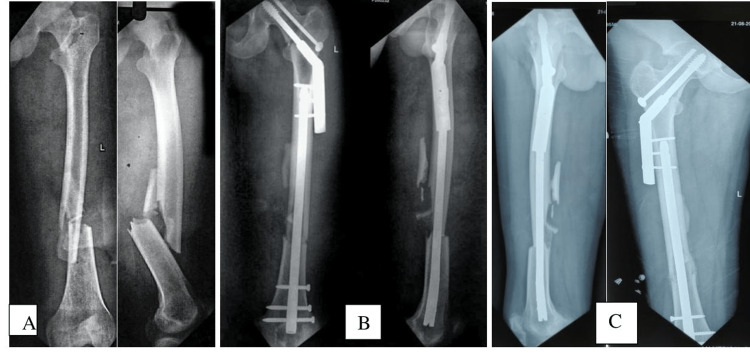
A) preoperative image; B) an immediate postoperative radiograph with good reduction and internal fixation with plate combinations; C) A four-month postoperative radiograph showing a union of the intertrochanteric and femoral shaft fractures

**Figure 3:**
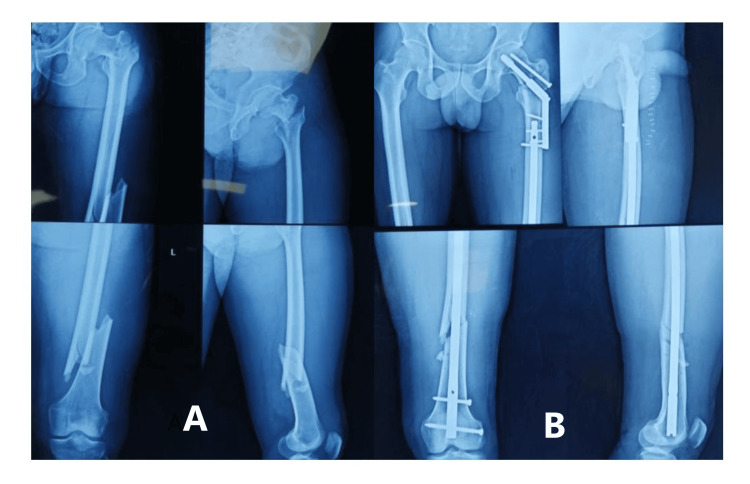
A) preoperative image; B) an immediate postoperative radiograph showing good reduction and internal fixation with plate combinations

**Figure 4:**
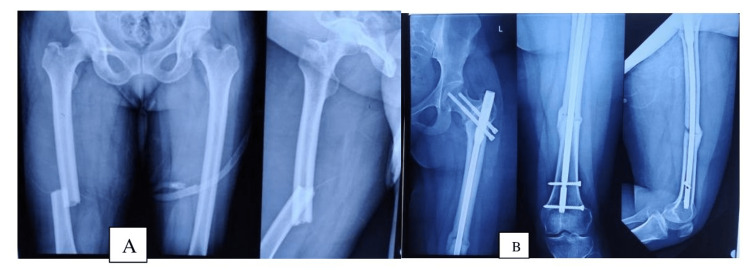
A) preoperative; B) a 20-week postoperative radiograph showing the union of the intertrochanteric and femoral shaft fractures

The preoperative and postoperative radiographs showing good reduction and union in the dual implant group are shown in Figure 2 and Figure 3 and the single implant group in Figure 4.

The mean duration of surgery in group I was 180.61+/-20.96 minutes, whereas in group II, it was 200.61+/-22.03 minutes. The surgical time was less in group I; however, the difference is not statistically significant (p=0.386) (Table 2).

**Table 2:** Clinical and functional outcomes of both groups

Items	Group I	Group II	p‐value
Age (years)	38.8 (17-63)	37.89 (21-65)	NS (0.8)
Male:female	15:1	16:2	NS (0.25)
Intertrochanteric fracture: AO‐classification			
31‐A1	4	4	
31‐A2	10	6	
31‐A3 31B2	2 0	5 3	
Femoral shaft fracture: AO-classification 32A	3	3	
32‐B	3	4	
32‐C1	5	5	
32‐C2	3	4	
32‐C3	2	2	
Average operative time (min)	180.61+/-20.96	200.61+/-22.03	0.38
Average intraoperative blood loss (mL)	490 (200-1000)	696 (500-1200)	0.04
Complications with proximal femoral fracture	Varus malunion 2 patients and delayed union in 1 patient	Deep infection 1 patient	
Union	16	18	NS (1.0)
Non-union	0	0	
Femoral shaft fracture			
Union	15	18	NS (0.4)
Non-union or delayed union	1	0	
Proximal femur fracture average union time (weeks)	20.68+/-6.3	18.67+/-6.5	NS (0.934)
Femoral shaft fracture average union time (weeks)	24.13+/-6.3	21.89+/-6.23	NS (0.368)
Functional outcome			NS (0.709)
Good	8	9	
Fair	7	8	
Poor	1	1	

Average blood loss was higher in group II compared to group I, and the difference was statistically significant (p=0.04) (Table 2). On clinical functional evaluation by Friedman-Wyman criteria [2], seven patients in group I had a fair outcome, eight patients had a good outcome and one patient had a poor outcome because the limitation in range of movement was more than 50 percent. Eight patients had a fair outcome, nine patients had a good outcome, and one patient had a poor outcome with a loss of range of motion of more than 50 percent in group II. Applying the Chi-squared test showed no significant difference in functional outcomes (Table 2).

Out of 18 patients in group II, one patient developed a deep infection, which was managed by debridement, antibiotic cement beads, and intravenous antibiotics. Out of 16 patients in group I, two patients had varus malunion of intertrochanteric fracture, and also one patient developed delayed union of shaft femur, which was managed by bone grafting (Table 2).

No patient developed non-union or avascular necrosis of the femoral head in any of the groups.

## Discussion

An ipsilateral combination of proximal femur and shaft femur fractures are uncommon fractures, which makes it very challenging to manage. It has now been well established that surgical fixation is the optimum treatment. One significant and unresolved subject is the order of tackling these fractures. Developing an ideal fixation strategy for such fracture is very controversial. At present, there are two schools of thought for managing these combination injuries. Either individualized treatment for both fractures or a single implant to manage both fractures in one go.

In our study, when a patient presented to us with an ipsilateral proximal femur fracture with a shaft femur fracture, first, we looked and managed the proximal femur fracture, and if the fracture involved a neck femur (AOOTA31B), then it was managed by dual implants except for basicervical (31B3), which can be managed by both dual implants or single implant like PFNA2. In our series, eight patients had a fractured neck of the femur; six out of eight were treated with DFN and DHS, and two patients (AOOTA31B3) with PFNA2. In almost all cases, maintaining anatomical neck alignment is a must regardless of whatever implant we use. Provisional or definitive fixation of the neck is done first before fixing the shaft, as it prevents further displacement of the neck and avoids further arterial damage to the neck femur. Bedi et al. (2009) [11] and Wang et al. (2012) [18] in their study also proposed fixation of the neck femur first then followed by shaft fixation. However, Park et al. in 2006 [12] advocated first fixation of the femoral shaft fracture and then fixing displaced femoral neck fractures to have better control over the shaft fragment while reducing the neck fracture. But even they came to the conclusion that the kind of femoral neck fracture and the surgeon's knowledge, with the selected treatment approach should be the primary determinants of the treatment strategy. Bucholz and Koldenhoven suggested the proximal fracture should be managed first. The femoral neck fracture should preferably be stabilized first, and a delay of five to six days does not affect the ultimate functional outcome. So previous literature and basic principles are the foundation of our initial strategy in our clinical algorithm to manage proximal femur fracture.

Next, if it was an intertrochanteric fracture of AOOTA Type 31A, then we looked for the level of shaft femur fracture (AOOTA32). If it was AOOTA32 at distal 1/3rd, the area of the wide medullary canal, then we managed by dual implants separately for both fractures like DFN or LCDCP for shaft and short PFN or DHS for proximal fracture. For the rest of the cases involving proximal 1/3rd and mid 1/3rd of the femoral shaft, both fractures were managed by a single implant like long PFN or PFNA2. While doing this, the proximal fracture was always reduced first, and reduction was temporarily held by K-wires.

In our study, the mean duration of the union of the proximal femur and shaft femur in group I is more or less equal to group II, and the difference is statistically insignificant. Wang et al. (2012) in their study found that both treatment methods achieve satisfactory functional outcomes in patients with ipsilateral intertrochanteric and femoral shaft fractures [18]. In contrast to our study, where the length of surgery was roughly equal in both groups, they recommended PFNA-long as the better option for the treatment of complex fractures due to its advantages of being minimally invasive, decreased per-operative blood loss, and attaining biological fixation of both of fractures with a single implant.

In our study, none of the patients developed a non-union or AVN in any group. Only two patients developed varus malunion, and only one patient developed delayed union of shaft femur in a single implant group, which was statistically insignificant. Bedi et al. (2009) reported that internal fixation with a single device resulted in a much greater risk of malreduction of either the neck or shaft fractures [11]. Vidyadhara and Rao reported a delayed union of shaft fracture in more than 50% of patients treated with a single implant [9]. Additional surgery was also needed in six cases, while in our patients, only one additional surgery in group II due to deep infection was needed. Similarly, Jain et al. reported a 20% incidence of femoral shaft non-union using single reconstruction nailing [10]. According to Alho et al. [7], the femoral shaft fracture was the primary determinant of the patients' final prognosis. Watson and Moed [21] also noted a higher rate of non-union of the shaft femur and more malunion in hip fractures. These high rates of complications in the aforementioned studies could be explained by the fact that they decided to use a single implant in all such fracture configurations. In the current study, by following the management protocol as described above (Figure 1), we were able to minimize complications like non-union, malunion, and avascular necrosis. This is also the reason for good to satisfactory functional outcomes in 32 out of 34 patients in our study, and the functional outcomes between the two groups are also comparable.

Our study has a few limitations. There was no randomization, and the follow-up was relatively short. Also, we couldn't comment anything about the timing of surgery as it was determined by many other factors like other concomitant injuries and the medical fitness of the patient. Also, ideally, to compare both the groups, it should be one combination of only a single combination of implants only in group II, and this may confound the interpretation of results. Another limitation of our study is carrying out many evaluations, which when applying student's T test, may have increased the level of error. However, we do not believe that these limitations contradict our findings because concurrent ipsilateral proximal femur and shaft of the femur fractures are uncommon, and randomization is not achievable in such fracture combinations.

## Conclusions

For concurrent ipsilateral diaphyseal and proximal femur fractures, both dual and single implants are equally effective alternatives if properly applied as per the authors' clinical algorithm. Implant selection primarily depends on the pattern of injury, and the authors' clinical algorithm can be a suitable guide for the selection of implants. This can help in the proper standardized treatment of such fractures with uniformly good clinical results for patients. This will also minimize complications such as non-union and repeat procedures to overcome such complications.
